# Coffee: Fuel for Your Day or Foe for Your Arteries

**DOI:** 10.3390/antiox13121455

**Published:** 2024-11-27

**Authors:** Mabel Buelna-Chontal

**Affiliations:** Department of Cardiovascular Biomedicine, National Institute of Cardiology, Ignacio Chavez, Mexico City 14080, Mexico; mabel.buelna@cardiologia.org.mx; Tel.: +52-55-732-911 (ext. 25704)

**Keywords:** cardiovascular disease, atherosclerosis, obesity, coffee consumption, cholesterol

## Abstract

Atherosclerosis, a major cause of cardiovascular diseases, is influenced by modifiable factors such as adiposity and blood cholesterol. Diet is crucial in these areas, particularly regarding antioxidant, inflammatory, and obesity effects. Coffee, a globally popular stimulant beverage, has garnered significant attention for its potential impact on cardiovascular diseases. Recent insights reinforce the need to re-examine the relationship between coffee consumption and atherosclerosis progression. Coffee’s complex composition includes polyphenols, renowned for their antioxidant and anti-inflammatory properties as well as potential weight-reducing effects. In addition, studies have demonstrated that certain coffee compounds such as chlorogenic acid, caffeic, p-coumaric, and ferulic acid can prevent atherogenesis by preventing the oxidation of low-density lipoproteins. Conversely, diterpenes, found in some coffee brews, can elevate cholesterol levels, posing a risk to coronary health. Notably, coffee intake has been shown to influence gut microbiota diversity, potentially contributing to anti-obesity effects. This review explores the insights from preclinical and clinical studies investigating the potential mechanisms through which coffee consumption may reduce the risk of atherosclerosis—highlighting the potential benefits of moderate filtered coffee consumption and the potential risks associated with excessive coffee consumption. Understanding this relationship is crucial for informing public health recommendations and guiding future research.

## 1. Introduction

Coffee is one of the most popular beverages consumed globally. According to the International Coffee Organization [1], the annual per capita consumption in the top coffee-consuming countries ranges from 15.4 to 22.3 kg. Coffee contains a complex mixture of compounds with a myriad of bioactive molecules. In addition, the coffee brewing method also influences beverage composition, mainly affecting the lipid profile [2]. Coffee contains phytochemicals with well-recognized antioxidant properties, as evidenced in animal models [3] and humans [4]. Extensive research has explored the antioxidant properties of coffee [5]. While coffee intake has not been linked to the development of coronary or carotid atherosclerosis [6,7], this review delves into the potential mechanisms through which coffee compounds may influence atherosclerosis progression, considering associated factors such as obesity and lipid profile. By examining the diverse bioactive molecules in coffee and their potential effects on cardiovascular health, this review aims to contribute to a better understanding of the role of coffee consumption in preventing atherosclerosis and related risk factors.

## 2. Coffee Composition

Coffee has a complex composition containing a diverse array of biologically active compounds. The specific composition can vary depending on the type of coffee bean such as *Coffea arabica*, *Coffea canephora*, and *Coffea liberica*. Key components include phenolic acids (chlorogenic acids, cafestol, and kahweol), alkaloids (caffeine and trigonelline), methylxanthines (caffeine, theobromine, and theophylline), and nicotinic acid (vitamin B3) [8]. Chlorogenic acid (CGA) stands out as a particularly potent antioxidant and is also found in other foods, for instance, wine, tea, apples, mushrooms, etc. [9]. The biological properties of coffee are primarily attributed to key compounds such as caffeine, cafestol, kahweol, ferulic acid, CGA, and trigonelline.

## 3. Bioactive Compounds in Coffee and Its Potential to Prevent Atherosclerosis

### 3.1. The Impact of Coffee Consumption on Obesity

Obesity is a well-established risk factor for atherosclerosis and coronary artery disease. Obesity is a condition that results from an imbalance between energy intake and energy expenditure, where excess energy in the form of triglycerides is stored in adipose tissue and other tissues. Moreover, obesity alters the morphology and composition of adipose tissue, leading to changes in adipokine secretion, which is tightly associated with low-chronic inflammation [10]. Adipocyte dysfunction tends to cause dyslipidemia, oxidative stress, and endothelial dysfunction, which are tightly related to atherosclerosis development [11]. Peroxisome proliferator-activated receptors (PPARs) are transcription factors particularly relevant during the complex mechanism involved in adipocyte differentiation. PPARs are activated by lipid ligands and are critical in energy homeostasis, metabolism, and inflammation [12]. The effects of coffee and its compounds on obesity have been investigated. A recent study demonstrated that caffeine and CGA, when loaded into solid lipid nanoparticles, can decrease the expression of PPAR-γ and CCAAT/enhancer binding protein alpha (c/EBP-α), adipogenic biomarkers in a 3T3-F422A preadipocyte cell line [13].

A detailed overview of the effects of coffee and its bioactive compounds is presented in Table 1. In this respect, the effect of coffee in the regulation of lipid metabolism, reducing the size and number of adipocytes, has been widely described in vitro [14,15,16]. Additionally, mice fed with a combination of CGA and caffeine (0.2%/0.03%) for 24 weeks exhibited decreased intraperitoneal adipose tissue, body weight, serum/hepatic of total cholesterol, triacylglycerol, and leptin levels. This response was associated with increased AMP-activated protein kinase (AMPK) expression and decreased PPAR-γ2 expression in the liver [17]. Likewise, coffee consumption (1% *w*/*w*) along with a high-fat diet for 10 weeks in a mouse model showed the attenuation of adipose tissue inflammation, a minor body mass gain, and an improvement in body fat distribution, mainly driven by PPAR-γ expression to promote adaptive thermogenesis related to a healthier fat distribution profile [18]. These outcomes from in vitro and animal studies suggest a potential reversible effect of coffee’s bioactive compounds on adipogenesis. This response involves the promotion of lipolysis by AMPK and adaptive thermogenesis driven by PPAR-γ, which reduces lipid accumulation in adipocytes, supporting their anti-obesity effects, and have been further evaluated in human studies.

Additionally, numerous human studies have examined the effect of coffee consumption on obesity. In particular, green coffee extract supplementation with high CGA content has been shown to reduce body weight and body mass index. A crossover placebo-controlled trial showed that 500 mg/day of green coffee bean extract supplementation during 1-week reduced body weight and the body mass index in healthy volunteers [19]. Likewise, a longer intervention study (8 weeks) with a higher dose of decaffeinated green coffee supplementation (800 mg/day, containing 186 mg of CGA) in patients diagnosed with metabolic syndrome reduced the body weight and body mass index [20]. Moreover, a clinical trial in overweight/obese patients with type 2 diabetes demonstrated the protective effects of 800 mg/day of green coffee extract for 10 weeks, leading to reductions in body weight, body mass index, systolic blood pressure, C-reactive protein (CRP), triglycerides, and higher high-density lipoprotein (HDL) levels [7]. However, coffee’s anti-obesity effects can be blunted by an excessive intake in specific populations with underlying health conditions. A 2-year follow-up study found a correlation between high coffee consumption and increased body adiposity in individuals with kidney transplant [21]. A 3-year follow-up study in elderly participants with metabolic syndrome demonstrated that moderate, but not high, coffee consumption was related to a reduction in total fat tissue, trunk fat, and visceral adipose tissue. However, heavy coffee intake was linked to unhealthier lifestyle habits among consumers, which could potentially counteract the beneficial effects of coffee on weight loss [22]. Overall, the previous findings support that coffee compounds can contribute to weight loss under specific conditions, with dosage playing a crucial role. Indeed, individual health conditions should be considered, as should significant limitations in clinical studies including short intervention periods, low sample size, and low female representation. Although more comprehension is needed to understand the complex relationship between coffee intake and weight loss, there is a new perspective on the increasing research field on gut microbiota and related health consequences. In the next section, the major effects of coffee consumption, improving the diversity of gut microbiota and exerting an anti-obesity effect, are analyzed.

#### Coffee Consumption and Gut Microbiota in Weight Loss

Coffee metabolism occurs in three phases: early absorption within the first 1–2 h in the stomach and small intestine, intermediate absorption between 4 and 8 h, and the late absorption phase in the large intestine after 8 h. This late absorption phase is particularly influenced by the gut microbiota [23]. Coffee is fermented by gut microbiota in the colon, which can regulate the bioavailability and biological activity of coffee polyphenols [24]. Moreover, coffee compounds including CGA and caffeine can exert beneficial effects on the intestinal microbiota, potentially leading to weight loss. A study showed that coffee consumption could have positive effects in high-fat fed rats including decreased body weight, lower adiposity, and improved gut microbiota diversity [24]. In another study using C57BL/6 male mice fed a high-fat diet, a 150 mg/Kg CGA solution for 20 weeks led to a loss of body weight and prevented subcutaneous and visceral weight gain, through its regulation of gut microbiota [25]. Of note, gut microbiota diversity has been well-established as a significant factor in atherosclerosis progression [26]. While green coffee extract intake did not directly decrease atherosclerotic lesions or serum lipids in ApoE−/− mice fed a high-fat diet for 14 weeks, it did result in reduced adiposity, weight gain, and improved gut microbiota diversity [27]. Furthermore, in an animal model of high-fat diet using Wistar rats, a freeze-dried coffee solution was found to increase the population of *Bifidobacterium* and improve HDL-C reverse cholesterol transport. However, despite these positive effects, it did not prevent weight gain in the experimental rats [28]. Of note, the significant limitations of these pre-clinical studies are the different dosages and treatment duration. Although most studies did not assess dosage according to the population’s average consumption, their findings support that coffee consumption through the action of CGA can improve gut microbiota diversity, potentially contributing to weight loss even under a high-fat diet. This highlights the importance of gut microbiota in mediating the cardiovascular effects of dietary compounds like coffee.

### 3.2. Coffee Consumption and Inflammation

Preclinical and clinical studies have addressed the effect of coffee compounds on inflammation. Experimental data in INS-1 cells exposed to 3 mM streptozotocin and preincubated with kahweol (2.5 and 5 μM) showed an increase in antioxidant enzymes and anti-inflammatory effects by downregulating nuclear factor kappa B (NF-κB) [29]. Indeed, kahweol has been shown to activate nuclear factor E2-related factor 2 (Nrf2) signaling, which is associated with antioxidant and anti-inflammatory responses [30]. In vitro studies have demonstrated anti-inflammatory properties of caffeine including its ability to suppress NF-κB activation in RAW264.7 cells stimulated with lipopolysaccharide (LPS) [31]. Additionally, caffeine, at various doses (0.019 mM, 0.102 mM, and 1.16 mM), has been shown to exert anti-inflammatory effects in peripheral blood mononuclear cells by downregulating the expression of pro-inflammatory genes such as signal transducer and activator of transcription 1 (STAT1), tumor necrosis factor (TNF)-α, interferon gamma (IFNG), and PPARG as well as cytokines including interleukins (IL)-8, IL-4, and IL-10 [32]. Treatment of RAW 264.7 cells with coffee pulp extract, CGA, and caffeine, in the presence of LPS, reduced NF-κB activation and the expression of inflammatory markers such as TNF-α, IL-6, inducible nitric oxide synthase (iNOS), cyclooxygenase-2 (COX-2), and prostaglandin E2 (PGE2) [33]. On the other hand, in a mouse model of high-fat diet-induced obesity, coffee intake (2% freeze-dried coffee or 2% green coffee extract) for 9 weeks decreased body weight gain and reduced the expression of inflammatory markers including activating transcription factor 3 (Atf3), Fos, and suppressor of cytokine signaling (Socs3) [34]. Furthermore, a study in C57BL/6 mice fed a high-fat diet found that consuming instant organic coffee (0.1% *v*/*v*) for 4 weeks prevented glucose intolerance, hypertrophy, and reduced macrophage infiltration, IL-6, and TNF-α levels, suggesting a decrease in adipose tissue inflammation [18]. While in vitro data support the potential of kahweol, caffeine, and CGA to suppress inflammatory mediators, the doses assessed may not be directly found in coffee beverages typically used by consumers. Regarding the animal studies, although a coffee extract was used instead of only specific components, the treatment duration was still too variable. Notwithstanding the limitations, these studies support the anti-inflammatory effects of coffee compounds.

Additionally, coffee and its constituents have also been shown to exhibit anti-inflammatory effects in human subjects. A study in subjects with and without coronary artery disease showed that acute caffeine ingestion (200 mg) decreased the CRP in plasma and improved brachial endothelial function [35]. Furthermore, two large cohorts of health professionals, with a follow-up between 9 and 14 years, demonstrated that regular coffee consumption, both caffeinated and decaffeinated, was associated with lower levels of inflammatory mediators such as CRP, leptin, and IL-6 [36]. Likewise, long-term filtered caffeinated and decaffeinated coffee consumption in healthy and type 2 diabetes women have been demonstrated to diminish inflammation, as indicated by lower levels of CRP, and prevent endothelial dysfunction, as assessed by a decrease in E-selectin [37]. In a placebo-controlled trial in cyclists, high-CGA coffee consumption (prepared using the Turkish method) for 2 weeks increased the antioxidant capacity but did not decrease post-exercise inflammation [38]. This may be due to CGA’s primary role as an antioxidant rather than an anti-inflammatory agent. A clinical trial conducted among habitual coffee drinkers with an elevated risk of type 2 diabetes demonstrated that increasing the filtered coffee doses (4 cups/day to 8 cups/day for 4 weeks each dose) had favorable effects only on the markers of subclinical inflammation, IL-18 and 8-isoprostane (8-IsoP), without adversely affecting proatherogenic lipids [39]. However, the duration of this study was too short, the dosage was considerably high, and most of the subjects were female (77%) and obese. Another clinical study found that moderate coffee consumption (3 cups per day for 8 weeks) of a soluble mixture of green and roasted coffee provided benefits for both healthy and hypercholesterolemic subjects. Coffee intake decreased the total cholesterol, low-density lipoprotein (LDL)-cholesterol, very low-density lipoprotein (VLDL)-cholesterol, and triglycerides, along with improved antioxidant capacity, decreased malondialdehyde (MDA), carbonylated groups, and decreasing CRP, which are associated with chronic inflammatory conditions [4]. Findings from clinical studies suggest that coffee compounds other than caffeine may be responsible for anti-inflammatory effects, and both acute and long-term consumption impact inflammatory status. Therefore, the anti-inflammatory properties of coffee compounds suggest a potential to reduce the risk for atherosclerosis since the attenuation of systemic inflammation has demonstrated significant reductions in secondary atherosclerotic cardiovascular disease; however, inter-individual differences should be considered.

#### Genetic Variations and Physical Activity Influences the Impact of Coffee Consumption on Inflammation

There are beneficial effects of coffee intake on inflammation and cardiovascular health; however, the influence of inter-individual differences should be considered including genetic variations and lifestyle habits. A study showed that coffee consumption triggered an anti-inflammatory response in individuals with the adenosine A2A receptor (ADORA2A) TT genotype after resistance exercise or in physically active subjects [40]. Another study examining peripheral blood mononuclear cells from healthy subjects before and after coffee consumption found that inflammatory markers exhibited a differential response among individuals, concluding that caffeine may be pro-inflammatory in some individuals [41]. Moreover, genetic polymorphisms in metabolic enzymes, such as cytochrome P4501A2 (CYP1A2) [42], adenosine receptors [43], and transcription factor Nrf2 [44], can account for individual variations in caffeine metabolism and response. Notably, heavy coffee consumers carrying the polymorphisms rs2472297-T and rs6968865-T have higher caffeine clearance due to the enhancement of CYP1A1 and CYP1A2 enzyme activity [45], potentially modulating their response to coffee consumption.

Additionally, lifestyle factors significantly influence the response to coffee intake. A cross-sectional study found that while habitual caffeine intake in healthy subjects could decrease the CRP in plasma, physical activity and sedentarism influenced coffee’s impact on inflammatory status [46]. Therefore, genetic variability and lifestyle factors, such as physical activity, can modulate individual responses to coffee consumption. These include anti-inflammatory effects and potential adverse effects like caffeine dependence, arrhythmias, anxiety, and sleep disturbances, which can impact cardiovascular health.

### 3.3. Coffee Extraction Method Influence the Lipid Profile

While coffee polyphenols offer protective effects against cardiovascular diseases and associated risk factors, coffee oils can have detrimental effects by raising the serum lipid levels [47]. This finding generates concerns about the potential impact of coffee intake on cardiovascular health, but the specific outcomes can vary depending on the coffee’s composition and the extraction method used [48,49]. Even though diterpenes are intrinsic compounds of the coffee lipid fraction, it is crucial to highlight that coffee extraction has a certain complexity and not only determines the coffee flavor profile, but also the physicochemical composition related to biologically active substances aside from caffeine such as lipid-soluble compounds [48]. For instance, coffee brewed using a French press typically has a low content of polyphenols and a high content of kahweol and cafestol, both found in the lipid fraction, while filtered coffee has the opposite profile [49]. Specifically, a high content of diterpenes such as cafestol and kahweol (around 4.43 mg/cup) has been found in unfiltered/boiled coffee from Indonesia, Scandinavian-boiled, Turkish, and French press, which can contribute to elevated cholesterol and triglyceride levels in the serum [50,51,52]. The regular consumption of Turkish coffee by individuals from a Jordanian population, which is high in cafestol and kahweol, has been shown to increase cholesterol, triglycerides, and LDL-C levels, suggesting a potential risk for cardiovascular diseases [50]. The effect on cholesterol metabolism associated with cafestol and kahweol is primarily due to a lower bile acid excretion and neutral sterols [53]. Among this study’s limitations, the diverse dietary habits of participants and the lack of control over coffee beverage additives may have influenced the results regarding lipid profiles. Another study demonstrated that the consumption of unfiltered coffee (Turkish method) increased the total cholesterol to HDL ratio in women with vitamin D deficiency, potentially increasing their risk of hyperlipidemia [54]. This finding supports that drinking boiled and unfiltered coffee may increase the serum lipid levels. However, the study’s limitations, such as the diverse coffee consumption dosage and lack of dietary data, should be considered when interpreting the outcomes.

**Figure 1:**
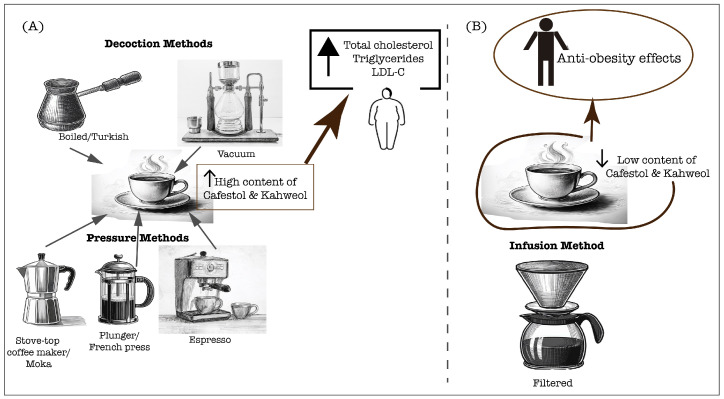
Coffee extraction methods significantly determine the physicochemical characteristics of the final brew, in particular, the content of cafestol and kahweol. These compounds can directly affect the lipid profiles of coffee consumers, which are crucial for cardiovascular health. (**A**) Unfiltered preparations, including decoction and pressure methods, lead to a high content of diterpenes, cafestol and kahweol that contribute to rise of serum lipids; (**B**) Filtered coffee preparation leads to a lower content of cafestol and kahweol, therefore allowing the anti-obesity effects associated with coffee consumption.

Conversely, paper-filtering coffee effectively removes most of the coffee oils, attenuating the cholesterol-raising effect and enabling the anti-atherogenic effects mediated primarily by coffee phenolic compounds, which will be further explored in this manuscript [51]. Moreover, in traditionally prepared coffee in Singapore and India, the content of diterpenes was negligible (0.09 mg/cup), which did not increase the serum cholesterol and triglyceride levels [52]. A study in the Taiwanese population demonstrated that the long-term consumption of at least five cups per week of plain black coffee or with additives, increased the HDL-C levels, which is crucial for cardiometabolic health through its antiatherogenic potential [55]. However, the preparation method was not indicated, and the additives’ variability should be considered in the lipid profile outcomes. Additionally, a 2-year follow-up study examining the long-term effects of coffee consumption on lipid profiles found that heavy consumption was associated with increased total cholesterol, triglycerides, and VLDL-C [56]. It is important to note that this study was conducted in a large cohort of Brazilians, and most participants typically drank filtered coffee, which is the traditional brewing method in Brazil. Moreover, a study by Svatun et al. [57] found that consuming three to five cups of espresso per day was associated with higher serum cholesterol levels in both men and women. Similarly, consuming more than six cups of boiled/plunger coffee daily increased the cholesterol levels in both genders. However, a high intake of filtered coffee (more than six cups per day) only elevated the serum cholesterol in women [57]. These findings suggest that high coffee consumption raises cholesterol levels, especially when unfiltered coffee is consumed. However, high filtered coffee consumption can also lead to high cholesterol levels, especially in women, suggesting a sex difference response. Furthermore, the limitations of this study include variability in diet and the added additives as well as the need for a dose–response curve to diterpene content. Therefore, the influence of coffee on the lipid profile is determined by the diterpene content, which is tightly related to the brewing method (Figure 1), and the dosage of coffee consumed is also crucial. These insights underscore the need for further research to understand how sex differences affect the relationship between coffee consumption and cholesterol levels.

### 3.4. Anti-Atherogenic Effects of Bioactive Compounds of Coffee

While elevated cholesterol, LDL, and triglycerides are well-established risk factors for atherosclerosis, the oxidation of LDL (ox-LDL) is essential in the progression of this disease. Ox-LDL is a pivotal step in atherogenesis, as outlined by the oxidation theory of atherosclerosis. While ox-LDL is a key factor, other components, such as lysophosphatidylcholine (lysoPC) and oxysterols, also contribute to atherosclerosis progression. LysoPC, a breakdown product of phosphatidylcholine, is an active component of ox-LDL. It plays a significant role in endothelial dysfunction and cardiovascular disease [58,59]. Oxylipins, derived from oxidized polyunsaturated fatty acids, and oxysterols, such as 7-ketocholesterol (7-KC), are also implicated in atherosclerosis due to their roles in inflammation and vascular function [60,61]. As previously mentioned, coffee is a rich source of antioxidants including alkaloids, flavonoids, and phenolic compounds. CGA and its metabolites, such as ferulic, isoferulic, and vanillic acids, have demonstrated potent antioxidant activity [62,63]. Given that ox-LDL is a contributing factor for the development of atherosclerotic plaques, the antioxidant properties of coffee compounds including phenolic acids have been shown to protect LDL from oxidation in both in vitro and in human studies.

An in vitro study demonstrated that coffee compounds including caffeic acid, 1-methyluric acid, and 1,3,7-trimethyluric acid could effectively prevent LDL oxidation [62]. Additionally, a study on healthy volunteers detected the presence of ferulic acid and caffeic acid in the plasma just one hour after consuming 400 mL of filtered coffee containing 420 mg of CGA. The early detection of these phenolic compounds in plasma upon acute coffee intake was associated with a heightened antioxidant capacity, with caffeic acid demonstrating a superior antioxidant profile. Furthermore, the in vitro study using plasma samples from the control subjects, when incubated with ferulic acid and caffeic acid (1–3 μM), showed an increase in antioxidant capacity and a delay in the oxidation of LDL [23]. Notably, caffeic acid was found to have a more pronounced effect on LDL oxidation resistance. Several clinical studies have addressed how coffee consumption may affect LDL oxidative susceptibility. A study of healthy male students aged 20 to 31 that consumed coffee for 7 days led to a significant decrease in total cholesterol, LDL-C levels, and lipid peroxidation markers and a significant reduction in LDL susceptibility to oxidation [64]. It is essential to consider the study’s limitations including its short-term coffee consumption (7 days) and its inclusion of only male young subjects in a small cohort. This effect was further supported by another study on samples from 10 healthy volunteers that showed that consuming 200 mL of filtered coffee increased the resistance of LDL to oxidation, likely due to the incorporation of coffee’s phenolic compounds, such as caffeic, p-coumaric, and ferulic acids, into LDL [65]. Furthermore, the moderate consumption of medium light roast or medium roast paper-filtered coffee for 8 weeks increased the antioxidant enzymes in the plasma of healthy volunteers but did not significantly reduce the ox-LDL levels [66]. These outcomes suggest an individual response variation to coffee consumption. Although the study comprised a small sample, the different nutritional and lifestyle habits among subjects also influenced the variations found.

Additionally, coffee polyphenols, such as CGA, are metabolized by the gut microbiota into smaller phenolic acids like dihydroferulic acid (DHFA). These metabolites can be detected in the plasma of coffee consumers at concentrations of up to 1 μM and have been shown to exhibit anti-inflammatory and antioxidant properties, potentially mitigating atherogenic progression [67]. As previously mentioned, oxylipins and oxysterols are among the oxidized lipids found in atherosclerotic plaques including 7-KC, making them target molecules to explore the effect of coffee compounds in the setting of atherosclerosis. A study on 74 subjects consuming two types of filtered coffee (high and low CGA content) for 8 weeks found that coffee with high CGA content decreased the oxylipin levels, lipid peroxidation markers, and inflammatory markers in plasma. Moreover, in the same study, in vitro experiments using THP-1 monocyte-derived macrophages exposed to 25 μg/mL ox-LDL further demonstrated that treatment with 1 μM DHFA led to a decline in ox-LDL uptake, mainly by the lower expression of ox-LDL receptors cluster of differentiation 36 (CD36), scavenger receptor class A (SR-A), lectin-like oxidized LDL receptor-1 (LOX-1) as well as lower reactive oxygen species (ROS) production and a decline in oxylipin levels [68]. These findings suggest that coffee consumption, particularly through CGA and DHFA, is a potential dietary intervention to reduce oxylipins, decreasing the risk for atherosclerosis. In a recent study, DHFA demonstrated its beneficial effects in cultured human macrophages exposed to inflammatory and oxidative stress conditions (50 μg/mL ox-LDL, 10 ng/mL LPS, or 20 μM 7KC). DHFA treatment (1 μM) decreased ROS, 8-IsoP, ox-LDL uptake, CD36 expression, and inflammatory mediators while simultaneously increasing IL-10 and prostaglandin PGE1, a potent vasodilator [68]. This study further supports the potential ability of DHFA, at the levels reported in plasma, to prevent atherogenic progression.

Another approach has been to study the effect of lysoPC since it is an active component of ox-LDL, as previously mentioned. An extensive study of 169 individuals found that filtered coffee consumption could decrease the lysoPC levels in plasma, primarily mediated by coffee polyphenols [69]. It should be considered that this was a cross-sectional study when interpreting the association between coffee consumption and lower levels of lysoPC. Moreover, subjects with an elevated risk of type 2 diabetes consuming 4 to 8 cups of paper-filtered coffee daily for 2 months reduced the lysoPC levels in plasma [70]. However, it should be considered that the effect found on lysoPC was related to the high coffee consumption (4 to 8 cups/day for 8 weeks) among the participants, and the cohort size was small in this study. The association of the decrease in lysoPC and ox-LDL by coffee consumption can partly be explained by the coffee polyphenols being transported in the plasma bound to LDL, potentially carrying antioxidants to the arterial intima and endothelial cells to protect LDL and phospholipids from oxidation as a consequence, thus also improving the antioxidant capacity [71]. Altogether, these findings are significant as they suggest a potential mechanism by which acute and long-term coffee consumption, primarily through the action of polyphenol compounds, may contribute to a minor oxidation susceptibility of lipids usually found in atherosclerotic plaques, thus preventing atherosclerosis progression. However, individual variations can influence this response.

**Table 1:** Evidence of the impact of coffee and coffee bioactive on adiposity, gut microbiota, antioxidant enzymes, inflammation, and lipid profile.

Coffee/Coffee Bioactive	Study Model	Main Findings	Study Details	References
Green and roasted coffee	Normocholesterolemic (*n* = 25) and hypercholesterolemic (*n* = 27) subjects aged 18 to 45 years	⇩ Total cholesterol ⇩ LDL-C ⇩ VLDL-C ⇩ Triglycerides ⇧ Plasma antioxidant capacity ⇩ MDA levels ⇩ Carbonylation ⇩ CRP	Moderate coffee consumption (3 cups per day) for 8 weeks.	[4]
Caffeine and CGA ⇧	3T3-F422A preadipocyte cell line	⇩ PPAR-γ expression ⇩ c/EBP-α	Caffeine 1 mM + CGA 0.5 mM loaded into solid lipid nanoparticles.	[13]
Caffeine and CGA	Female ICR mice	⇩ Adipose tissue ⇩ Body weight ⇩ Total cholesterol (serum and hepatic) ⇩ Triglycerides ⇩ Leptin levels ⇧ AMPK activation ⇩ PPAR-γ2 liver expression.	Diet containing: 0.2% CGA 0.03% caffeine for 24 weeks.	[17]
Instant organic coffee	C57BL6 male mice	Improved glucose metabolism ⇩ Adipose tissue inflammation ⇩ Hypertrophy ⇩ Macrophage infiltration ⇩ IL-6, TNF-α ⇧ Adaptive thermogenesis ⇧ Mitochondrial biogenesis	High-fat diet + consumption of instant organic coffee (0.1% *v*/*v*) for 4 weeks.	[18]
Green coffee bean extract	Individuals, over the age of 18, (*n* = 103)	⇩ Body weight Lipid profile improvement	500 mg/day green coffee extract. Supplementation for at least 1 week to 8 weeks.	[19]
Decaffeinated green coffee	Patients diagnosed with metabolic syndrome, aged 18–70 years (*n* = 43)	⇩ Body weight ⇩ Body mass index	8 weeks of decaffeinated green coffee supplementation (800 mg/day, containing 186 mg of CGA).	[20]
Green coffee extract	Overweight/ obese patients with type 2 diabetes (*n* = 44)	⇩ Body weight ⇩ Body mass index ⇩ Systolic blood pressure ⇩ C-reactive protein ⇩ Triglycerides ⇧ HDL levels	800 mg/day green coffee extract supplementation for 10 weeks	[7]
Coffee	Kidney transplant recipients aged 49.5 years (*n* = 170)	⇧ Body adiposity (central adiposity) Lower muscle quality	Median coffee consumption 200 mL/day 2 years follow-up.	[21]
Coffee	Individuals with metabolic syndrome (*n* = 1483)	⇩ Total fat tissue ⇩ Trunk fat ⇩ Visceral adipose tissue	Moderate coffee consumption (1–7 cups/week) 3 years follow-up.	[22]
Acute coffee consumption (400 mg CGA)	In vitro and ex vivo experiments on plasma from healthy volunteers (*n* = 20) after drinking coffee	⇧ Antioxidant capacity of plasma Prevention of LDL oxidation	Acute coffee consumption containing 420 mg of CGA (400 mL of coffee).	[23]
Instant coffee	High-fat fed rats (Male Sprague Dawley)	⇩ Body weight ⇩ Adiposity ⇧ Insulin resistance ⇩ *Firmicutes* (F)-to-*Bacteroidetes* ratio and *Clostridium Cluster* XI ⇧ *Enterobacteria*	Instant caffeinated coffee (20 g/L) for 10 weeks.	[24]
CGA	C57BL/6 male mice fed a high-fat diet	⇩ Body weight ⇩ Subcutaneous and visceral weight ⇧ Short chain fatty acid producers (*Dubosiella*, *Romboutsia*, *Mucispirillum*, and *Faecalibaculum*) ⇧ *Akkermansia*	150 mg/Kg CGA solution for 20 weeks.	[25]
Green coffee extract	Apo−/− mice fed antiatherogenic diet	⇩ Adiposity ⇩ Weight gain ⇩ Inflammatory infiltrate in adipose tissue Improved microbiota diversity ⇧ *Desulfovibrio* ⇧ *Mogibacteriaceae*	Green coffee extract 220 mg/Kg for 14 weeks.	[27]
Freeze-dried coffee solution	Wistar rats fed high-fat diet	⇧ *Bifidobacterium* spp. ⇧ HDL-C reverse cholesterol transport ⇩ II1b mRNA Did not improve weight gain	Freeze-dried coffee solution at a dose of 0.39 g/100 g for 8 weeks.	[28]
Kahweol	INS-1 cells	⇩ NF-κB ⇧ Antioxidant enzymes (Hemeoxygenase-1) ⇧ p-AKT ⇧ BCL-2	Cells were exposed to 3 mM streptozotocin and pre-incubated with 2.5 and 5 μM kahweol.	[29]
Kahweol	AREc32 cells	⇧ Nrf2	0.02 and 30 μM kahweol.	[30]
Caffeine	RAW264.7 cells	⇩ NF-κB ⇩ p-p38MAPK	Cells were exposed to 1 μg/mL LPS and treated with caffeine (0, 100, 400, 800, 1000, and 1200 μM).	[31]
Caffeine	Peripheral blood mononuclear cells isolated from 3 healthy individuals	⇩ STAT1 expression ⇩ TNF expression ⇩ IFNG expression ⇩ PPARG expression ⇩ IL-8, IL-4, IL10, and TNF-α levels	Caffeine (0.019 mM, 0.102 mM, and 1.16 mM).	[32]
Coffee pulp extract/CGA/caffeine	Raw 264.7 cells	⇩ TNF-α, IL-6, iNOS, COX-2, and PGE2 expression ⇩ NF-κB activation ⇩ MAPK signaling	Cells were stimulated with 1 μg/mL LPS and treated with 1000 μg/mL coffee pulp extract, 13.38 μg/mL CGA, and 3.82 μg/mL caffeine.	[33]
Coffee/Green coffee	C57BL6 male mice	⇩ Body weight ⇩ Mesenteric fat weight ⇩ Atf3, Fos, and Socs3 ⇩ Hsp70	High-fat diet 2% freeze-dried caffeinated coffee, decaffeinated coffee, or green coffee for 9 weeks.	[34]
Caffeine	Subjects with (*n* = 40) and without coronary artery disease (*n* = 40)	⇩ CRP in plasma Improvement in brachial endothelial function	200 mg acute caffeine ingestion.	[35]
Caffeinated and decaffeinated coffee	*n* = 15,551 women (nurse’s health study) and *n* = 7397 men (health professionals)	⇩ CRP ⇩ Leptin ⇩ IL-6 ⇩ C-peptide ⇩ Estrone, total estradiol, free estradiol ⇧ Adiponectin	Regular coffee consumption; Follow-up between 9 and 14 years.	[36]
Filtered coffee	Healthy women (*n* = 730) and women with type 2 diabetes (*n* = 663) aged 43–70 years	⇩ CRP Prevent endothelial dysfunction ⇩ E-selectin	Regular caffeinated and decaffeinated coffee consumption. Follow-up of 14 to 15 years.	[37]
High-CGA coffee	Cyclist subjects: men (*n* = 10), women (*n* = 5) aged 19 to 51 years	⇧ Antioxidant capacity in plasma It did not decrease post-exercise inflammation	High-CGA coffee consumption (300 mL/day) for 2 weeks. Coffee was prepared using the Turkish method. Participation in a 50-Km cycling time trial.	[38]
Filtered coffee	Habitual coffee drinkers (*n* = 47) younger than 65 years with elevated risk of type 2 diabetes	⇩ IL-18 ⇩ 8-Isoprostane ⇧ Adiponectin ⇧ Caffeine in serum ⇧ CGA in serum ⇧ Caffeic acid metabolites in serum ⇧ HDL ⇩ LDL/HDL ratio	First month: no coffee Second month: 4 cups/day Third month: 8 cups/day.	[39]
Caffeine/coffee	Resistance-trained Iranian men (*n* = 15) around 21 years old. Russian healthy physically active subjects (*n* = 134) aged 28 to 31 years	⇩ Myeloperoxidase ⇩ Acetylcholinesterase Association of *ADORA2A* gene polymorphism with anti-inflammatory effects of caffeine	6 mg/Kg acute caffeine consumption before resistance exercise. Regular coffee intake in the physically active subjects.	[40]
Coffee/caffeine	Peripheral blood mononuclear cells isolated from 8 healthy individuals	⇩ Inflammatory markers in some individuals ⇧ inflammatory markers in some individual	Cells were isolated before and after coffee consumption (3 capsules of coffee containing 165 mg caffeine). Exposed to 1 μg/mL LPS and 5 μg/mL phytohemagglutinin. Cells were treated with 200 ng/mL caffeine in vitro.	[41]
Caffeine	Healthy subjects: men (*n* = 112) and women (*n* = 132) aged 18 to 55 years	⇩ CRP in plasma ⇩ Body fat total and visceral ⇧ Adiponectin ⇧ Il-10 ⇩ IL-6, TNF-α	Habitual caffeine intake.	[46]
Coffee	Individuals (*n* = 109) aged 22 to 70 years	⇧ Total cholesterol ⇧ Triglycerides ⇧ LDL-C ⇧ VLDL-C	Regular coffee consumption (Turkish method and instant coffee).	[50]
Coffee	Women with vitamin D deficiency (*n* = 270) aged 18 to 65 years	⇧ Total cholesterol/HDL ratio	Turkish coffee consumption during 3 previous months. Moderate consumption (1–2 cups/day). High consumption (≥3 cups/day); 150 mg caffeine per cup.	[54]
Coffee	Healthy volunteers (*n* = 3000)	Filtered coffee: ⇩ Serum cholesterol ⇩ Triglycerides Unfiltered coffee: ⇧ Serum cholesterol ⇧ Triglycerides	Filtered and unfiltered coffee consumption (1–5 cups/day).	[52]
Coffee	Healthy volunteers (*n* = 1272) over the age of 30.	⇧ HDL-C levels	Regular plain black coffee consumption (5 cups per week). Follow-up of 13 years.	[55]
Filtered coffee	ELSA-Brazil cohort (*n* = 4732)	⇧ Total cholesterol ⇧ Triglycerides ⇧ VLDL-C ⇧ Triglyceride-rich lipoprotein particles	Regular high consumption of filtered coffee (more than 3 cups/day).	[56]
Coffee	Tromø study in northern Norway (*n* = 21,083) aged 40 years	⇧ Total cholesterol levels	Espresso coffee 3 to 5 cups per day. Boiled/plunger coffee more than 6 cups per day.	[57]
Caffeic acid, 1-methyluric acid, and 1,3,7-trimethyluric acid	In vitro and ex vivo study on plasma from healthy individuals	Prevention of LDL oxidation by copper	0.5 μM caffeic acid, 3 μM 1,3,7-trimethyluric acid, 30 μM 1-methyluric acid, caffeic acid.	[62]
Coffee	Healthy male volunteers aged 20 to 31 (*n* = 11)	⇩ Total cholesterol ⇩ LDL-C ⇩ MDA ⇩ LDL oxidation	Coffee intake, 24 g total per day for 1 week	[64]
Filtered coffee/caffeic acid	Ex vivo and in vitro experiments in plasma from healthy volunteers (*n* = 10)	⇩ LDL oxidation Incorporation of caffeic, *p*-coumaric, and ferulic acids into LDL	Coffee consumption (200 mL) In vitro: 1, 10, 100 nmol/L caffeic acid incubated with isolated LDL from healthy subjects.	[65]
Filtered coffee	Healthy volunteers (*n* = 20)	⇧ SOD ⇧ Catalase ⇧ GPx Did not reduce ox-LDL levels	482 ± 61 mL/day medium light roast or medium roast paper-filtered for 4 weeks.	[66]
DHFA	Culture human macrophages	⇩ ROS production ⇩ 8-Isoprostane ⇩ Ox-LDL uptake ⇩ CD36 expression ⇩ inflammatory mediators (TNF-α, IL-6, and IL-17) ⇧ IL-10 ⇧ PGE_1_	THP-1 monocyte-derived macrophages were exposed to 50 μg/mL ox-LDL, 10 ng/mL LPS or 20 μM 7KC treated with 1 μM DHFA.	[67]
Filtered coffee with high content of CGA and low content of kahweol and cafestol/DHFA in in vitro experiments.	Subjects (*n* = 74) aged between 20 and 60 years. In vitro experiments in THP-1 monocyte-derived macrophages	⇩ Oxylipin levels in plasma ⇩ Lipid peroxidation markers ⇩ Inflammatory markers No significant differences in ox-LDL levels in plasma In vitro data: ⇩ Ox-LDL uptake ⇩ CD36 expression ⇩ SR-A expression ⇩ LOX-1 expression ⇩ ROS production ⇩ Oxylipin profile	Consumption of coffee A containing 787 mg CGA (*n* = 24), coffee B containing 407 mg CGA (*n* = 25), 400 mL/day for 8 weeks. In vitro experiment: 25 μg/mL ox-LDL, 1 μM DHFA, and 1 μM phenolic acid.	[68]
Coffee (high content of polyphenols)	Healthy subjects aged 20 years or older (*n*= 169)	⇩ Plasma LysoPC levels.	Low coffee consumption (≤100 mL/day), high coffee consumption >100 mL/day).	[69]
Filtered coffee	Habitual coffee drinkers (*n* = 47)	⇩ Plasma LysoPC levels	First month: No coffee consumption. Second month: 4 cups of paper-filtered coffee/day. Third month: 8 cups of paper-filtered coffee/day.	[70]

CGA, chlorogenic acid; PPAR-γ, peroxisome proliferator-activated receptor-gamma; AMPK, AMP-activated protein kinase; LysoPC, lysophosphatidylcholine; SOD, superoxide dismutase; GPx, glutathione peroxidase; ox-LDL, oxidized-low density lipoproteins; DHFA, dihydroferulic acid; CD36, ox-LDL receptors cluster of differentiation 36; SR-A, scavenger receptor class A; ROS, reactive oxygen species; IL, interleukins; 7KC, 7-ketcholesterol; HDL, high-density lipoproteins; LDL, low-density lipoproteins; VLDL, very-low density lipoproteins; MDA, malondialdehyde; CRP, C-reactive protein; NF-κB, nuclear factor kappa B; Nrf2, nuclear factor erithroid-2 related factor 2; p-AKT, phosphorylated protein kinase B; LPS, lipopolysaccharide; STAT1, signal transducer and activator of transcription 1; TNF, tumor necrosis factor; IFNG, interferon-gamma; PPARG, peroxisome proliferator-activated receptor-gamma; iNOS, inducible nitric oxide synthase; COX-2, cyclooxygenase-2; PGE2, prostaglandin E2; Atf3, activating transcription factor 3; Socs3, suppressor of cytokine signaling; Hsp70, heat shock protein 70. ⇩ Refers to a lower/decrease; conversely ⇧ it indicates, an increase.

## 4. Additional Considerations

The effect of coffee consumption on cardiovascular health may be influenced by factors such as the extraction method, dosage, long-term effects, genetic variants, lifestyle habits, and underlying health conditions. Regarding dosage, moderate coffee consumption is generally defined as consuming no more than 3 cups/day (accounting for 183–583 mg/serving of caffeine), and more than 4 cups/day is considered excessive consumption [72]. According to Surma [72], a common brewed coffee typically comprises around 125 mL per serving, and a cup of espresso contains approximately 25 mL.

On the other hand, although beyond the scope of this review, the potential health risks related to excessive coffee consumption should also be considered, such as arrhythmias, caffeine dependence, anxiety, and insomnia, to name a few. In this regard, when high doses of coffee are consumed, more caffeine modulates the inhibition of phosphodiesterase, raising the cytosolic calcium concentration and causing atrial arrhythmias [73]. As a result, non-habitual or high-coffee consumption is related to a high risk of arrhythmias [72]. In addition, caffeine exerts its stimulant effects by blocking adenosine receptors, resulting in elevated cortisol, epinephrine, and dopamine levels, causing alertness, arousal, and anxiety symptoms [74]. Indeed, the stimulant properties of caffeine and its ability to modulate neurotransmitters such as dopamine contribute to its addictive potential. Furthermore, habitual caffeine consumption can lead to tolerance (i.e., a higher dosage is needed to promote the intended effects), and withdrawal symptoms can be experienced [75]. Caffeine-associated stimulant effects are also related to sleep disturbances, especially when consumed during the 6 h before sleep [74]. Altogether, these side effects are mainly associated with high caffeine intake, and it is essential to note that they may affect overall well-being.

## 5. Conclusions

Findings from in vitro or animal studies have allowed for an understanding of the specific effects of coffee compounds. Although limited by factors like dosage, which is not always equivalent to that regularly consumed by humans, or because only a specific compound of coffee was assessed, they offer a better comprehension of the mechanisms and particular action of bioactive compounds such as CGA and diterpenes; these studies provide a foundation for understanding the effects of coffee on obesity, the biodiversity of gut microbiota, inflammation, lipid profile, and anti-atherogenic potential. According to the evidence reviewed here, moderate coffee consumption (up to 3 cups/day) can be beneficial in preventing atherosclerosis progression. However, special attention should be paid to the brewing method, as diterpenes in boiled and unfiltered coffee may elevate the lipid levels in consumers, which can be a risk for cardiovascular diseases. Furthermore, although individual responses to coffee consumption can be influenced by genetic variations, the potential risks associated with excessive coffee consumption are associated with caffeine including caffeine dependence, anxiety, arrhythmias, and sleep disturbances. These insights underscore the need for further studies to better comprehend the complex interplay between coffee components and human health.
